# Complete genome sequence of *Microbacterium* sp. strain UB-LE1, isolated from Teutoburger Forest soil in Germany

**DOI:** 10.1128/mra.00443-26

**Published:** 2026-06-22

**Authors:** Bianca Laker, Michael Thomas, Prisca Viehoever, Levin J. Klages, Tobias Busche, Karsten Niehaus, Andrea Bräutigam, Marion Eisenhut

**Affiliations:** 1 Computational Biology, Faculty of Biology, Bielefeld University 98894, Bielefeld, Germany; 2 Center for Biotechnology (CeBiTec), Bielefeld University 9167, Bielefeld, Germany; 3 Medical School OWL, Bielefeld University 9167, Bielefeld, Germany; 4 Proteome and Metabolome Research, Faculty of Biology, Bielefeld University 98894, Bielefeld, Germany; 5 Genetics and Genomics of Plants, Faculty of Biology, Bielefeld University 98894, Bielefeld, Germany; DOE Joint Genome Institute, Berkeley, California, USA

**Keywords:** *Microbacterium*, xanthan degradation

## Abstract

We report the complete genome sequence of *Microbacterium* sp. strain UB-LE1, a xanthan-degrading bacterium isolated from Teutoburger Forest soil in Bielefeld, Germany. The circular genome is 3,410,522 bp with 70.46% G + C content.

## ANNOUNCEMENT

*Microbacterium* species are gram-positive, aerobic, rod-shaped bacteria found in diverse environments (1–4). Here, we report the complete genome of *Microbacterium* sp. strain UB-LE1, capable of utilizing xanthan as the sole carbon source, as outlined in our related work (5).

Approximately 1 g of topsoil was collected from the Teutoburger Forest, Bielefeld, Germany (52.01387°N, 8.48334°E; 4 April 2023), and transported on ice. The sample was mixed with 2.5× (weight/volume) phosphate-buffered saline (pH 7.0) and homogenized for 30 min. After centrifugation (500 × *g*, 5 min), each of the 12 flasks containing 100 mL M9 minimal medium with 0.5% xanthan (Jungbunzlauer, Switzerland) as the sole carbon source for xanthan-degrader enrichment was individually inoculated with 100 µL of supernatant. Cultures were incubated at 180 rpm and 28°C for 120 h. The culture with the strongest viscosity reduction and highest OD600 was selected. The enrichment culture was serially diluted (10^−4^), spread-plated on M9–xanthan agar, and incubated at 28°C for 48 h. After plating on LB medium, an orange colony was isolated and designated as UB-LE1. UB-LE1 was stored as a glycerol stock at −80°C.

For taxonomic classification, genomic DNA was extracted from 30 mg wet weight biomass, obtained from the orange colony streaked on LB medium, using the NucleoSpin Microbial DNA kit (Macherey-Nagel, Germany) for gram-positive bacteria. The 16S rRNA gene was amplified with primers 27F (5′-AGAGTTTGATCMTGGCTCAG-3′) and 1492R (5′-TACGGYTACCTTGTTACGACTT-3′) (6) using DreamTaq PCR master mix (Thermo Fisher Scientific, USA) and gel-purified using the MinElute kit (Qiagen, Germany) according to the manufacturer’s instructions. It was Sanger-sequenced with an Applied Biosystems 3730xl DNA Analyzer. BLASTN analysis against the 16S rRNA (Bacteria and Archaea) database (7) showed the highest similarity to *Microbacterium testaceum* (NCBI RefSeq NR_026163.1), confirming genus assignment.

For whole-genome sequencing, genomic DNA was applied for library preparation using the SQK-LSK114 ligation kit with NBD114-96 native barcoding without DNA sharing or size-selection (Oxford Nanopore Technologies [ONT], UK). Sequencing was performed on a GridION X5 with an R10.4.1 flow cell (ONT). Basecalling with Dorado v7.1.4 using model dna_r10.4.1_e8.2_400bps_sup@v4.2.0 (ONT) yielded 41,067 reads with an *N*_50_ of 11,162 bp (Table 1). Default parameters were used except where noted. Reads were trimmed with cutadapt v4.8 with parameters “fwd: -j 32 -e 0.2 -O 15 --trimmed-only -g AAGGTTAANNNNNNNNNNNNNNNNNNNNNNNNCAGCACCT -, rev: -j 32 -O 15 -e 0.2 -a AGGTGCTGNNNNNNNNNNNNNNNNNNNNNNNNTTAACCTTAGCAAT -m 50” (8) and assembled using Flye v2.9-b1774 with parameters --meta -m 2000 --read-error 0.02 --keep-haplotypes (8–10). Circular overlaps were trimmed by Flye. For polishing, reads were mapped with Minimap2 v2.26 (11), variants were called with BCFtools v1.18 (12), and the most abundant base per strand was selected. Genes were predicted with Prokka v1.14.5 (13). Completeness was assessed with benchmarking universal single-copy orthologs (BUSCO) v5.8.3 using microbacterium_odb12 (1,023 BUSCOs) (14). Genome-based taxonomic analysis was performed using the Type (Strain) Genome Server (TYGS) (15).

**TABLE 1:** Sequencing and assembly statistics for *Microbacterium* sp. strain UB-LE1

Parameter	Data
Raw genomic sequencing reads	
No. of reads	41,067
Total length (bp)	368,447,565
*N*_50_ (bp)	11,162
Genome sequence	
No. of sequences	1
Length (bp)	3,410,522
GC content (%)	70.46
Genome coverage (×)	108
Gene annotation	
Total no. of genes	3,163
No. of protein coding genes	3,103
No. of rRNAs	9
No. of tRNAs	50
No. of tmRNAs	1
BUSCO results (%)	
Complete	99.2
Single copy	99.1
Duplicated	0.1
Fragmented	0.4
Missing	0.4

**Fig 1:**
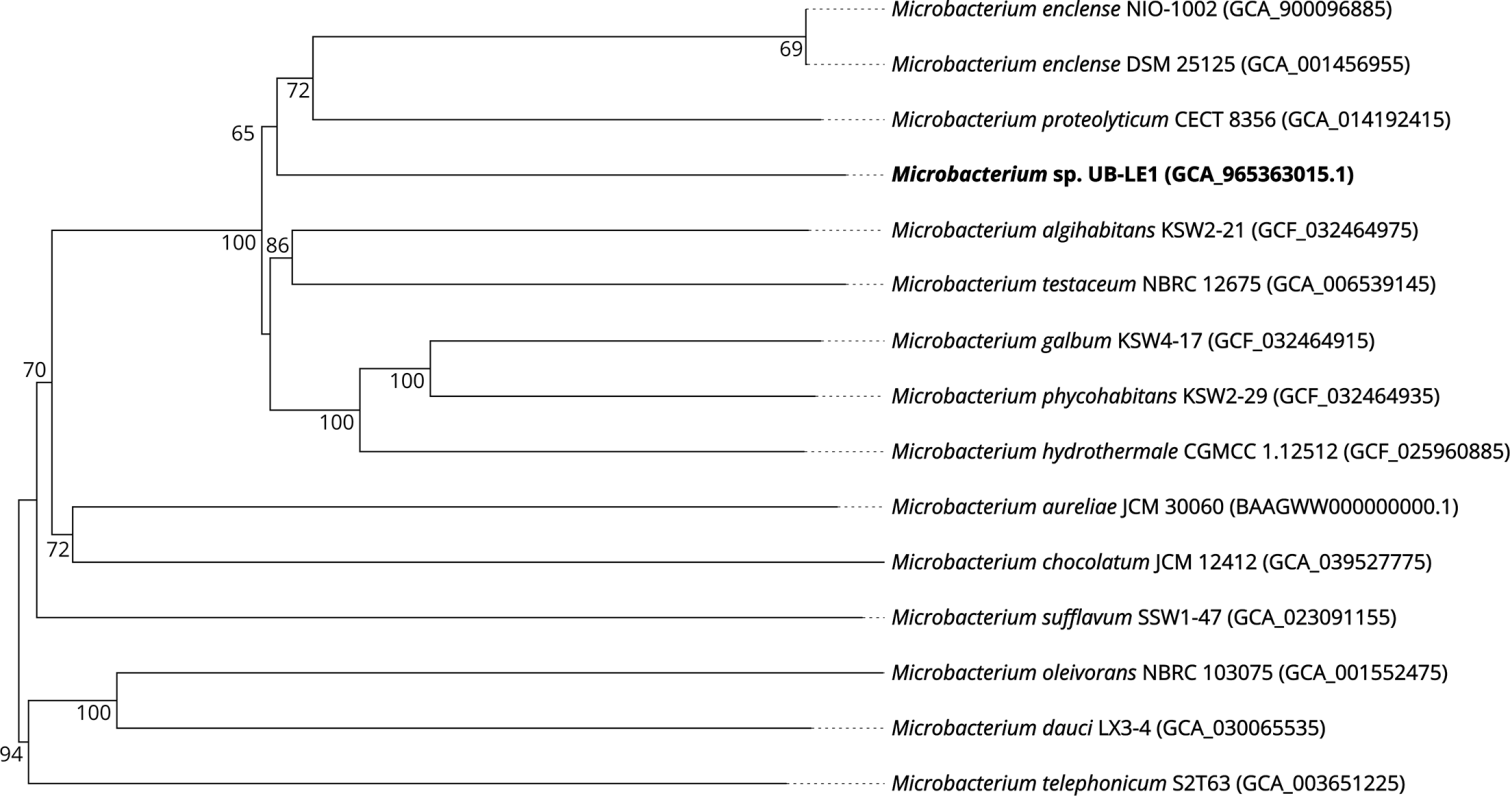
Species tree based on whole-genome sequences. Phylogenetic analysis of *M*. sp. UB-LE1 and closely related Microbacteria based on the TYGS server and the TYGS type strain database. Reference genomes were selected by the TYGS pipeline using a combination of whole-genome similarity with MASH and 16S rRNA gene sequence comparisons via BLAST. The whole-genome-based tree was inferred using the Genome BLAST Distance Phylogeny (GBDP) method implemented in TYGS and reconstructed with FastME v2.1.6.1 to generate a balanced minimum-evolution tree with Subtree Pruning and Regrafting (SPR) post-processing (16). Branch support was calculated from 100 pseudo-bootstrap replicates, the tree was midpoint-rooted, and branch lengths are scaled to the GBDP distance formula d5 (17).

The genome assembly consists of one circular chromosome of 3,410,522 bp, 70.46% G + C, 108× coverage, and 3,163 genes (Table 1). BUSCO completeness was 99.2%. Phylogenetic analysis highlights the divergence of UB-LE1 from other *Microbacterium* species (Fig. 1).

## Data Availability

The *M.* sp. UB-LE1 strain is deposited in the DSMZ under DSM 120304 and in the BCCM/LMG under LMG 34097. The genome assembly, gene annotation, and raw reads are available in GenBank/ENA under BioProject accession number PRJEB89987. The GenBank/ENA accession number for the raw reads is ERR15095669, whereas the accession number for the genome assembly and annotation is GCA_965363015.1. The accession number for the 16S rRNA sequence is OZ262651.
